# 2D CoOOH Sheet-Encapsulated Ni_2_P into Tubular Arrays Realizing 1000 mA cm^−2^-Level-Current-Density Hydrogen Evolution Over 100 h in Neutral Water

**DOI:** 10.1007/s40820-020-00476-4

**Published:** 2020-07-02

**Authors:** Shucong Zhang, Wenbin Wang, Feilong Hu, Yan Mi, Shuzhe Wang, Youwen Liu, Xiaomeng Ai, Jiakun Fang, Huiqiao Li, Tianyou Zhai

**Affiliations:** 1 Guangxi Key Laboratory of Chemistry and Engineering of Forest Products, Guangxi University for Nationalities, Nanning, 530008 Guangxi People’s Republic of China; 2 State Key Laboratory of Material Processing and Die and Mould Technology, and School of Materials Science and Engineering, Huazhong University of Science and Technology, Wuhan, 430074 Hubei People’s Republic of China; 3 State Key Lab of Advanced Electromagnetic Engineering and Technology, Huazhong University of Science and Technology, Wuhan, 430074 Hubei People’s Republic of China

**Keywords:** Large-scale hydrogen production, Mass transport, 2D adaptive material, Interfacial charge modulation, Multiscale coordinated regulation

## Abstract

Water electrolysis at high current density (1000 mA cm^−2^ level) with excellent durability especially in neutral electrolyte is the pivotal issue for green hydrogen from experiment to industrialization. In addition to the high intrinsic activity determined by the electronic structure, electrocatalysts are also required to be capable of fast mass transfer (electrolyte recharge and bubble overflow) and high mechanical stability. Herein, the 2D CoOOH sheet-encapsulated Ni_2_P into tubular arrays electrocatalytic system was proposed and realized 1000 mA cm^−2^-level-current-density hydrogen evolution over 100 h in neutral water. In designed catalysts, 2D stack structure as an adaptive material can buffer the shock of electrolyte convection, hydrogen bubble rupture, and evolution through the release of stress, which insure the long cycle stability. Meanwhile, the rich porosity between stacked units contributed the good infiltration of electrolyte and slippage of hydrogen bubbles, guaranteeing electrolyte fast recharge and bubble evolution at the high-current catalysis. Beyond that, the electron structure modulation induced by interfacial charge transfer is also beneficial to enhance the intrinsic activity. Profoundly, the multiscale coordinated regulation will provide a guide to design high-efficiency industrial electrocatalysts.

**Electronic supplementary material:**

The online version of this article (10.1007/s40820-020-00476-4) contains supplementary material, which is available to authorized users.

## Highlights

- The 2D CoOOH sheet-encapsulated Ni_2_P into tubular arrays electrocatalytic system with expediting mass transport, structural stability, and tuned electron was conceptually proposed.
- The designed electrocatalysts realize expectant 1000 mA cm^−2^-level-current-density hydrogen evolution in neutral water for over 100 h.

**Electronic supplementary material:**

The online version of this article (10.1007/s40820-020-00476-4) contains supplementary material, which is available to authorized users.

## Graphical abstract

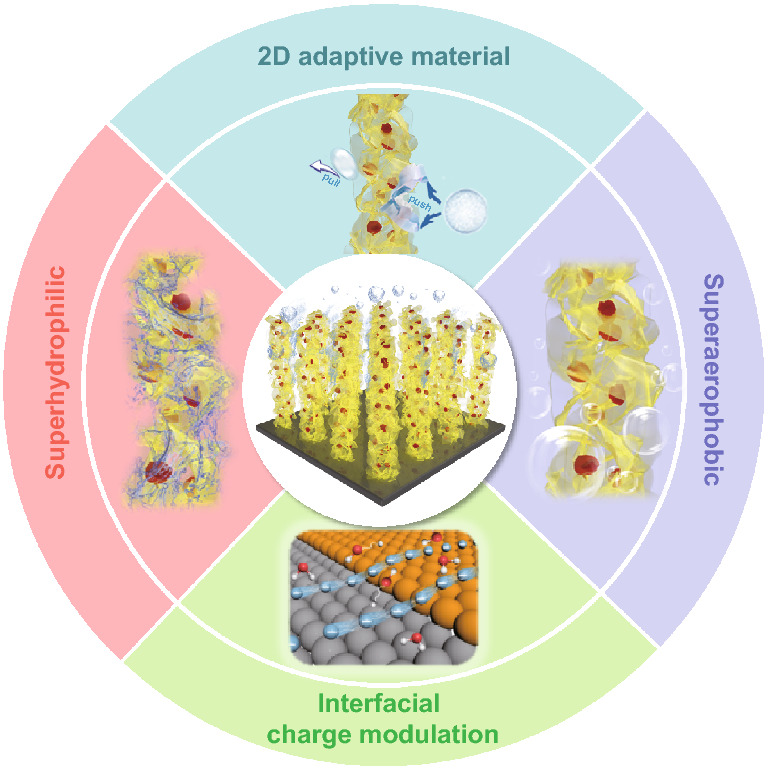

## Introduction

The blueprint for the hydrogen economy has been imagined that intermittent electric energy is used to drive water electrolysis for hydrogen production to be subsequently combusted in an engine or transformed into serviceable electricity in a fuel cell [1–3]. As an important part of the hydrogen cycle system, electrochemical water splitting in industry is disappointingly sluggish because of expensive noble metal catalysts and unsatisfactory activity [4]. In this regard, although a series of non-noble electrocatalysts and design strategy are springing up experimentally, most of them are mainly focusing on the overpotential and current density tends to be on 100 mA cm^−2^ level, unsatisfactory for the commercial requirements [5]. For practical industrial large-scale H_2_ production, high current density (1000 mA cm^−2^ level) and durability (over 100 h) are the key indicator, whereas raised insufficient attention [6]. More meaningful but challenging, the efficient operation of catalysts under neutral conditions not only can resist the corrosion of equipment in acidic and alkaline media currently used in industry, but also can help us understand the new mechanism of H_2_O molecule decomposition and hydrogen production [7]. Therefore, it is of high desire to develop excellent electrocatalytic hydrogen evolution catalysts with high current and long cycle for industrial large-scale hydrogen production under neutral conditions, so as to greatly promote the development of laboratory to commercial use in this promising field.

Fully examining the catalytic process, high intrinsic activity is the prerequisite of hydrogen evolution at large current, which requires the structural design at atomic scale to regulate the local electronic structure to lever the efficient decomposition of water molecules [8, 9]. In addition, rapid catalytic reactions at large currents will result in swift consumption of electrolyte and large production of hydrogen bubbles. At the moment, mass transfer becomes rate-determining step, requiring an immediate supply of electrolyte and rapid efflux of bubbles [10, 11]. In this context, superaerophobic and superhydrophilic under the regulation of solid–liquid–gas micro/nano-interface could be a valid model. It is noteworthy that high mechanical strength is required for the impact and drag of electrolyte convection and hydrogen bubble breaking and separating on the catalyst. Previous efforts have demonstrated several catalyst systems including 2H Nb_1+*x*_S_2_, Ni_2_P nanoarray, and NiMoN@NiFeN for high-current operations [12–14]. Although impressive progress, there are few reports on that give comprehensive attention to the synergistic enhancement of mass transport, intrinsic activity, and especially mechanical stability. Overall, it is expecting to implement multiscale structural regulation for acquiring integration of multifunctional units to achieve large-scale electrolysis of water to produce hydrogen.

On this background, most intuitively, the array configuration grown directly on the current collector may be an ideal model because its large area bonding contact can ensure smooth electron migration and resist catalyst shedding during long-time electrocatalysis [15, 16]. Meanwhile, the pioneering works from Gao’s, Sun’s, and Jin’s groups indicated the superaerophobic and superhydrophilic in the surface of array configuration, which will be expected to fast electrolyte recharge and bubble overflow at the superhigh-current catalysis [10, 17, 18]. Furthermore, to resist the impact of bubble overflow and rupture on the catalyst under high current for obtaining long-term stability [19], the microscopic configurations of individual structural elements in the array configuration need to be considered and designed. The 2D ultrathin sheet with elastic continuum can be considered as a time-honored adaptive material, whose softness can resist impact by releasing stress and has good mechanical stability [20, 21]. Therefore, it is possible to realize both expediting mass transport and structural stability by using 2D sheet as the basic unit and assembling it into an array through stacking and wrapping. On this basis, breaking the chemical bonds of water molecules to obtain efficient intrinsic catalytic performance is the premise of large-scale electrolysis of water to produce hydrogen, which requires it to be further designed from the atomic scale. In this connection, coupled with an active HER catalyst (such as MoS_2_, CoSe_2_, and Ni_2_P) as a hydrogen acceptor and oxygen evolution reaction (OER) catalyst (such as CoOOH, Co(OH)_2_, and NiO) as a hydroxyl acceptor into Janus heterojunction may be an effective strategy for dramatically facilitating decomposition of water molecules [22–28]. Banking on the above analysis, it is instructive to integrate “array-2D sheet-Janus heterojunction” into a catalytic system for large-scale hydrogen production.

**Scheme 1:**
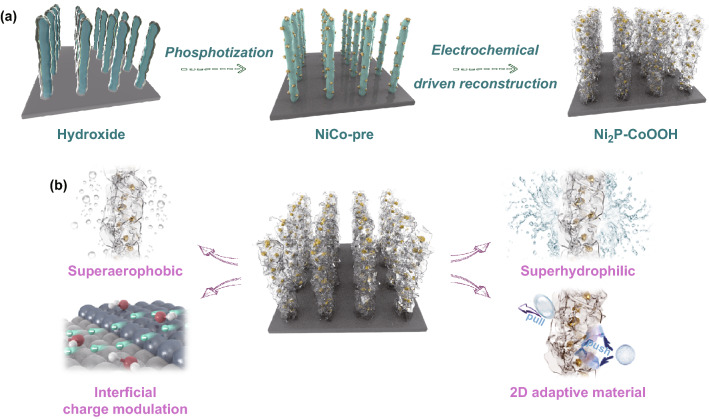
Schematic illustration of design thought. **a** Synthetic and multiscale coordinated regulation strategy. The designed 2D sheet-encapsulated tubular arrays catalysts were fabricated by in situ electrochemical driven reconstruction from bimetallic core–shell precursor NiCo-pre. **b** Multiscale structural regulation for acquiring a catalytic structure model with expediting mass transport, structural stability, and tuned electron

Mindfully, the designed catalytic system involves a variety of interfaces (catalyst and collector, 2D sheet-to-sheet, Janus heterojunction), in which the bonding requirements between these interfaces are diversiform. Specifically, catalyst and collector, Janus heterojunction need to be tightly bonded, but 2D sheet-to-sheet may need some gaps to ensure the transfer of material. In the experimental construction, how to synthesize the aggregation with different functional units according to the requirements in order is challenging. Herein, taking metallic CoOOH and Ni_2_P as active OER and hydrogen evolution reaction (HER) catalyst as example, the 2D CoOOH sheet-encapsulated Ni_2_P into tubular arrays electrocatalytic system with “one stone and three birds” was conceptually proposed and acquired by electrochemical driven reconstruction (Scheme 1). On the one hand, this in situ synthesis process can overcome the obstacle of lattice mismatch between CoOOH and Ni_2_P and form close-coupled heterojunction with chemical bonds, on the other hand, the generated bubbles in electrochemical driven reconstruction may act as the space steric hindrance guarantee rich gap of 2D sheet-to-sheet. As expected, we realized expectant 1000 mA cm^−2^-level-current-density hydrogen evolution in neutral water and 100 mA cm^−2^-level-current-density in seawater for over 100 h, which may push the electrolysis of water to produce hydrogen from laboratory to industry. Profoundly, the multiscale coordinated regulation permitted by this work will provide a guide to design high-efficiency electrocatalysts with a large current and excellent durability.

## Experimental Section

### Materials Synthesis

NiCo-pre was synthesized *via* a hydrothermal method. First, the carbon fibers (CF) substrates were pre-cleaned with diluted HCl solution, acetone, deionized (DI) water, and ethanol separately to remove the oxide layer of CF. After that, 0.05 mol L^−1^ Co(NO_3_)_2_·6H_2_O, 0.1 mol L^−1^ NH_4_F, and 0.25 mol L^−1^ urea were transferred into a 20-mL Teflon-lined stainless-steel autoclave with the pre-cleaned CF (2 × 2.4 cm^2^) placed against the wall of Teflon vessel, and the sealed autoclave was further kept at 120 °C in an electric oven for 12 h, then the Co(CO_3_)_0.5_OH nanowires were succeed synthesized on the carbon fibers. After cooling naturally to room temperature, it was taken out from the autoclaves, alternately washed with DI water and ethanol for several times, and dried at 60 °C overnight. Then, 0.0075 mol L^−1^ Ni(NO_3_)_2_·6H_2_O and 0.25 mol L^−1^ urea were dissolved in 20-mL Teflon-lined stainless-steel autoclave. Co(CO_3_)_0.5_OH/Ni(OH)_2_ nanowires were prepared by the second hydrothermal reaction at 100 °C for 6 h. After that, the Co(CO_3_)_0.5_OH/Ni_2_P nanowires (called NiCo-pre) were fabricated by annealing the Co(CO_3_)_0.5_OH/Ni(OH)_2_ nanowires in a home-built tube furnace at 300 °C for 1 h, with NaH_2_PO_2_ as phosphorus source placed at the upstream position and nitrogen gas as the carrier gas. Subsequently, the Ni_2_P–CoOOH was synthesized by the electrochemical transformation in 1 M KOH solution with a voltage of 1.6 V for 10 h. In this process, the Co(CO_3_)_0.5_OH nanowires have been transformed into CoOOH nanosheets wrapping on the Ni_2_P.

### Characterizations

The powder X-ray diffraction (XRD) patterns were recorded on a Rigaku MiniFlex 600 X-ray diffractometer with Cu Kα radiation (*λ* = 1.5406 Å). The Raman investigation was performed on a laser confocal Raman spectroscopy (Horiba-Jobin Yvon HR800, WITec) using a 532 nm laser as the excitation source. The morphology and microstructure of the as-prepared samples were examined by SEM (FEI quanta650) and high-resolution transmission electron microscopy (HRTEM, FEI Tecnai G2 F20 S-TWIN). The X-ray photoelectron spectroscopy (XPS) was performed on a Kratos AXIS Ultra DLD X-ray photoelectron spectrometer with a monochromatic X-ray source (Al Kα *hυ* = 1486.6 eV).

### In Situ Operation

The in situ operation was performed on SEM which equipped the mechanical arm (MM3A, Kleindiek nanotechnik) and the tungsten probe. Firstly, we use SEM to find a suitable single nanotube, which the probe will not touch other nanotubes. After that, the probe is moved directly to the down-side of the selected nanotube. Then, the probe is controlled to move to the up-side of nanotube perpendicularly in a constant speed. Meanwhile, a settled volt is applied on the probe to push the selected nanotube. Thus, the nanotube will bend while the probe approaching. And the bending angle increases with the push of probe. When the bending angle reaches to the settled value, the probe goes backward in the same way while the release of probe volt. Meanwhile, the nanotube quickly springs back to its initial position. After that, a second round of probe push is carried out in the same way to reach a larger bending angle. This procedure was repeated for several times, and the recoil time and the resilience value of nanotube are recorded. Through the above-mentioned operations, the macroscopic mechanical properties of nanotubes can be observed visually.

The specific parameters for SEM test are: vacuum degree is low than 8 × 10^−3^, voltage is in the range of 10–20 kV, electron beam spot is in the range of 3.4–4.5 nm and the working distance is more than 10 mm. The mechanical arm was operated by adjusting the XYZ axis according to the position of probe in the field of view.

### Electrochemical Measurements

The electrochemical measurements were taken on electrochemical workstation (Biologic VSP, Claix, France) using a three-electrode system. The obtained sample served directly as the working electrode, while graphite rod and saturated calomel electrode (saturated KCl solution) were used as the counter electrode and the reference electrode, respectively. The comparison samples were prepared by drop-coating Pt/C and RuO_2_ catalyst ink on CF (2.5 mg cm^−2^). The Pt/C catalyst ink was prepared by homogeneously dispersing 5 mg Pt/C (20 wt%) and 10 μL of 5 wt% Nafion solution in 1 mL water/ethanol (1:3 v/v) solution. The electrolyte (1 M KOH) was prepared by dissolving 56 g of high purity KOH pellets (AR, ≥99.5%, Aladdin Co., Ltd.) in 1 L DI water. The 1 M PBS electrolyte was prepared via the mixture of 1 M NaH_2_PO_4_ and 1 M Na_2_HPO_4_ solutions with a ratio of 38:62, and which was calibrated to pH = 7 by a pH meter. The seawater was purchased from Chuang Feng automation technology co., LTD. All potentials measured were converted to reversible hydrogen electrode (RHE), using the following equation: *E* (vs. RHE) = *E* + 0.241 + 0.059pH, where the *E* is the applied potential. Electrochemical performances for overall water splitting were tested in a two-electrode system. The prepared Ni_2_P–CoOOH and Ni–Fe LDH samples were used as cathode and anode, respectively. For comparison, noble metal catalysts Pt/C and RuO_2_ were also used as cathode and anode under the same conditions. The amounts of produced H_2_ and O_2_ were collected via a commercial two-electrode water splitting setting using a drainage method. H_2_ amount for Ni_2_P–CoOOH ||Ni–Fe LDH was tested *via* a Hoffman apparatus setup. In order to detect the H_2_ amount more accurately, methyl orange was added into the electrolyte (0.05 mg mL^−1^) for observation. The Faradaic efficiency was calculated by comparing the measured and theoretically produced amounts of H_2_. Polarization curves were recorded at the scan rate of 2 mV s^−1^. The corresponding Tafel plots were obtained by fitting the linear portion between the overpotential (*η*) and log current (log *j*), using the equation *η* = *b*log (*j*) + *a*, where *b* is the Tafel slope. Electrochemical impedance spectroscopy (EIS) measurements were taken at a potential perturbation of 5 mV amplitude in the range of 10 kHz to 0.1 Hz. Double-layer capacitance (*C*_dl_) data were collected *via* cyclic voltammetry curves, which measured at the scanning rates from 20 to 120 mV s^−1^. The current density differences (Δ*j* = *j*_*a*_ − *j*_*c*_) were plotted against scan rates, the linear slope of which, twice of the values for *C*_dl_ was used to estimate the ECSA. All the measured potentials were calibrated with 85% i*R* compensation if not explicitly specified, where *R* is the solution resistance.

### DFT Calculations

Simulated model in this work of a 2 × 2 supercell of the Ni_2_P (110) and CoOOH (001) surface including 4 atomic layers. All calculations were performed by density functional theory (DFT) implemented in the Vienna Ab initio Software Package (VASP 5.3.5) code by Perdew–Burke–Ernzerhof (PBE) and the projected augmented wave (PAW) methods [29–32]. The plane-wave cutoff energy was 400 eV and the Brillouin zone of the surface unit cell was sampled by Monkhorst–Pack (MP) grids. The Ni_2_P (110) and CoOOH (001) surfaces were determined by using 4 × 3 × 1 and 4 × 4 × 1 Monkhorst–Pack grid [33]. The convergence criterion was 10^−5^ eV and 0.01 eV Å^−1^ for the electronic self-consistent iteration and force, respectively. Using the climbing image nudged elastic band (CI-NEB) method to confirm the transition states, and the transition states only one imaginary frequency along the reaction coordinates [34–36]. Introducing a vacuum layer of 12 Å to avoid interactions between periodic images. The adsorption energy (E_ads_) of the surface species is defined by E_ads_ = E_total_ − E_surface_ − E_species_. Thereinto, E_total_ represents the total energy of the adsorbed species with catalyst surface, E_surface_ represents the energy of the empty surface, E_species_ represents the energy of the species in the gas phase.

## Results and Discussion

### Structural Characterization

**Fig. 1:**
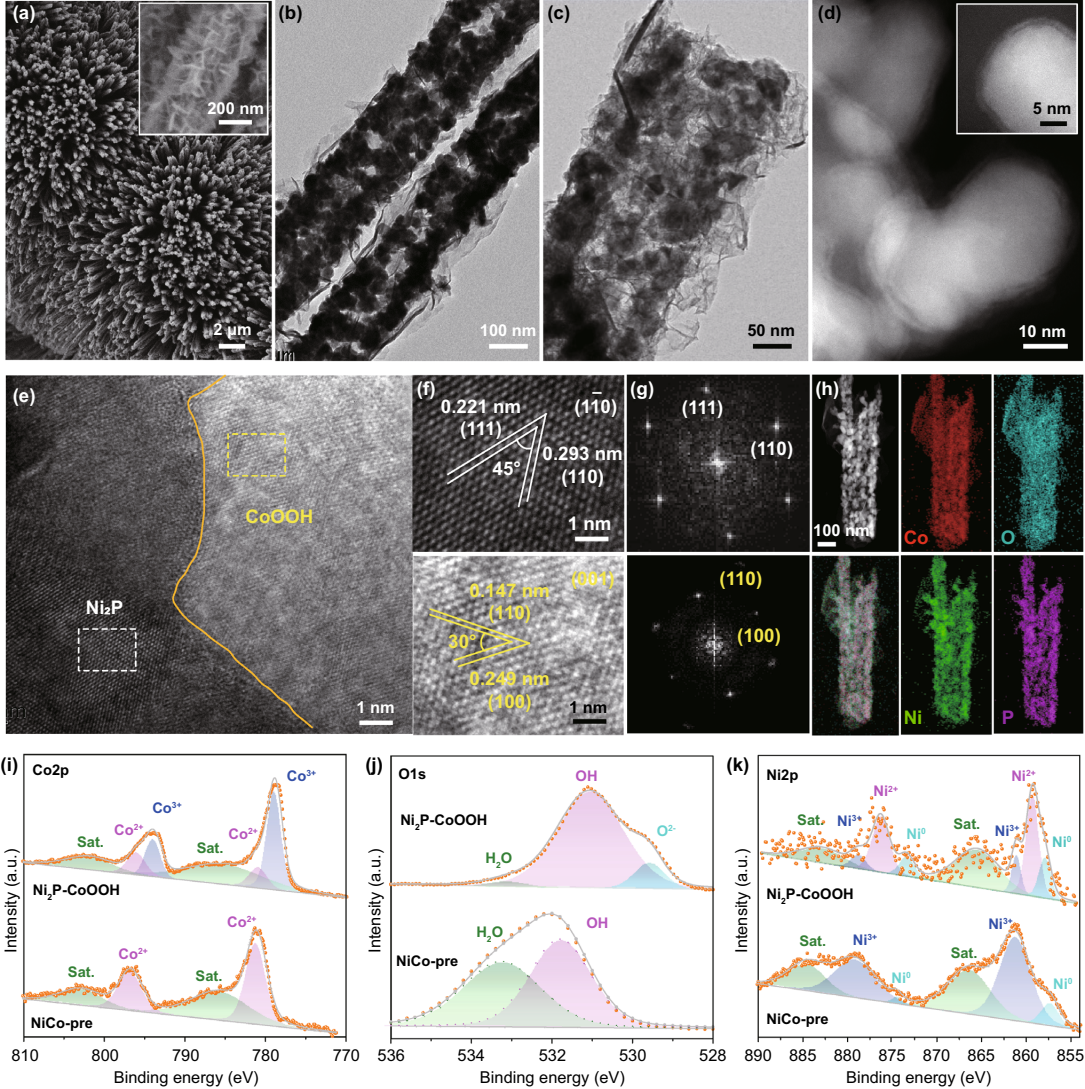
Structural characterization of designed catalysts. **a** SEM images, **b** low-magnified TEM, and **c** high-magnified TEM images of the Ni_2_P–CoOOH. **d** STEM image of the Ni_2_P–CoOOH, the inset is local information. **e** HRTEM image of the Ni_2_P–CoOOH. **f**, **g** Enlarged view of heterointerfaces between Ni_2_P and CoOOH lattice fringes (right: FFT). **h** STEM–EDX elemental mapping of the Ni_2_P–CoOOH. **i–l** XPS of the synthesis NiCo-pre and Ni_2_P–CoOOH

The designed 2D sheet-encapsulated tubular arrays catalysts were successfully fabricated by in situ electrochemical driven reconstruction from bimetallic core–shell precursor NiCo-pre (Fig. S1). The XRD pattern and the Raman spectrum indexed to Ni_2_P and CoOOH in the derived tubular arrays (Fig. S2), suggesting the successful electrochemical reconstruction [37]. Notably, the metallic behavior of Ni_2_P and CoOOH guarantees smooth electron migration during the electrocatalytic process. More insight into the microstructure of the obtained tubular arrays is provided by SEM and TEM. It is obvious that the Ni_2_P–CoOOH nanotube are vertically rooted on the carbon fibers, and the magnification of single unit displays staggered sheets covering the surface of nanotube (Fig. 1a). Namely, the superficial Co precursors in NiCo-pre precursors evolve into CoOOH lamellar structure under the action of a high current, which can overcome the obstacle of lattice mismatch between CoOOH and Ni_2_P and form close-coupled heterojunction with chemical bonds. Furthermore, at high-magnified TEM images (Fig. 1b, c), nanotubes are interwoven with typical stacked and staggered ultrathin nanosheets, as a consequence, maintaining hollow configuration with abundant infiltration gap. This tiered sheets on surface not only endow the abundant edges which reduce the adhesive force of gas, but also facilitate release of the bending stress which is crucial for stability at high current density. Additionally, scanning transmission electron microscopy (STEM) image shows that nanoparticles are tightly riveted and wrapped by supple ultrathin sheets on the surface of a hollow rod (Fig. 1d), revealing the strong coupling for effective electron modulation. Moreover, the high-resolution transmission electron microscopy (HRTEM) was performed to explore the atomic structure and bonding situation between nanosheets/particles interface (Figs. 1e, S3). Specifically, the well-resolved lattice fringes with inter-planar spacing of 0.221 and 0.293 nm can be unambiguously assigned to the (111) and (110) crystal planes of Ni_2_P, which are consistent with the previous XRD pattern. Moreover, the lattice fringes of 0.147 and 0.249 nm match well with the (110) and (100) crystal plane of CoOOH, revealing the coexistence of Ni_2_P and CoOOH in the heterojunctions (Fig. 1f). Fourier transformation patterns (Fig. 1g) further confirm the above conclusion. Overall, the above structure indicates the successful synthesis of the 2D CoOOH sheet-encapsulated Ni_2_P into tubular arrays, namely expectant “array-2D sheet-Janus interface” configuration. The schematic of synthetic process and formation mechanism of nanotubes are schematically shown in Figs. S4 and S5, respectively. Notably, this in situ electrochemical activation proposed in this work is also a new strategy for reconstructing tightly bonded heterogeneous hierarchical structures on the scale of morphology and surface chemistry. In addition, the elemental mapping and line scanning results (Figs. 1h, S6) demonstrate that Co and O are homogeneously distributed in nanosheets, while Ni and P are concentrated locating in nanoparticles, and the directed distribution of all elements illustrates the in situ formation of the Ni_2_P–CoOOH. The chemical composition and oxidation state of the Ni_2_P–CoOOH surface were further probed by XPS. Specifically, the wide-scanning XPS spectrum of the Ni_2_P–CoOOH samples reveal the coexistence of C, O, Co, Ni, and P elements on the nanowire surface (Fig. S7a). The fine scanning Co 2*p* spectrum (Fig. 1i), consisting of two major peaks of Co 2*p*_3/2_ (778.7 eV) and Co 2*p*_1/2_ (793.7 eV), can be indexed as Co^3+^, and the two declining peaks at 780.8 and 796.9 eV are assigned to Co^2+^, which demonstrate the formation of CoOOH [38]. Besides, the peaks at 529.6 and 531.1 eV are ascribed to the Co–O–Co bond and the Co–OH bond in O 1 *s* spectrum, which further confirm the above conclusion (Fig. 1j) [39]. In the Ni 2*p* spectrum (Fig. 1k), the two weaken peaks at 861.4 and 878.8 eV can be assigned to nickel phosphate, maybe it has dissolved in the electrochemical reconstruction process. The peaks at 857.3 and 874.5 eV are assigned to Ni_2_P [40], and two spin–orbit doublets of Ni 2*p*_3/2_ (860.2 eV) and Ni 2*p*_1/2_ (876.7 eV) can be indexed to Ni^2+^, which indicates the forming of Ni–O bond through the strong interatomic coupling at interface. The peaks at 133.5 and 135.4 eV originate from P 2*p* which can be assigned to Ni_2_P (Fig. S7b) [13]. In general, based on the above structural characterization, the designed “array-2D sheet-Janus heterojunction” model was successfully synthesized in the form of 2D CoOOH sheet-encapsulated Ni_2_P into tubular arrays by in situ electrochemical driven reconstruction.

### Electrochemical Characterization

**Fig. 2:**
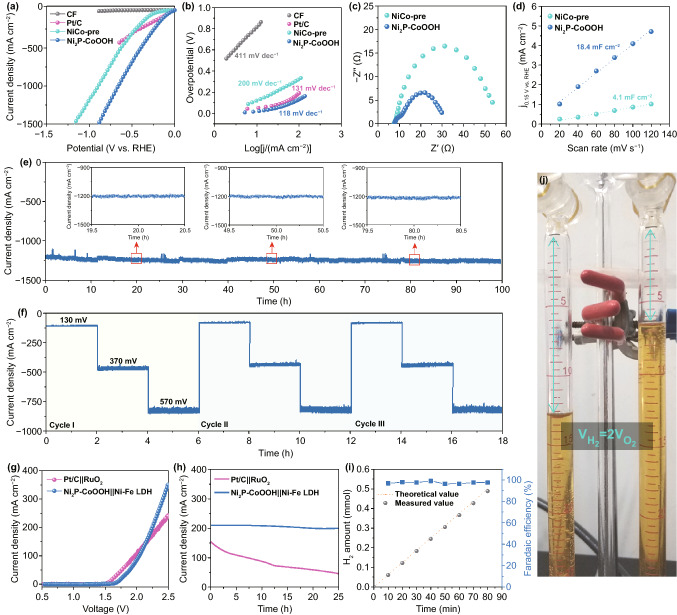
Electrocatalytic properties in 1 M PBS electrolyte. **a**, **b** Polarization curves and the corresponding Tafel plots. **c** EIS at a voltage of 0.2 V versus RHE. **d** Double-layer capacitance (*C*_dl_). **e** Chronopotentiometric curves of Ni_2_P–CoOOH at − 0.75 V versus RHE, the insets are local information. **f** Multi-chronoamperometric response curve of Ni_2_P–CoOOH. **g** Polarization curves for overall water splitting. **h** Chronopotentiometric curve of water electrolysis at 2.25 V. **i** H_2_ amount for Ni_2_P–CoOOH||Ni–Fe LDH at a fixed current density. **j** Optical photograph of the measured setup of the Hoffman apparatus

A neutral-effective electrocatalyst has appeared beneficial environmental benignity and bright application prospects, however, insufficient breakthrough research in this field has been reported thus far owing to the large ohmic loss and low ionic concentration [41, 42]. Herein, the electrochemical measurements of designed Ni_2_P–CoOOH were taken for evaluating the HER activity in neutral solution. CF, Pt/C, and NiCo-pre as the comparison samples were also investigated. The reaction of Ni_2_P–CoOOH electrode starts immediately on the application of potential, which with obviously initially advantage (Fig. S8). While the increasing of potential, it proceeds rapidly to achieve a current density over 1500 mA cm^−1^ (Fig. 2a). It is noteworthy the unprecedented current density of Ni_2_P–CoOOH in neutral electrolyte surpasses most of the transition metal-based catalysts previously reported (Table S1), which will be a candidate for large-scale hydrogen production. Further assessment of the HER activity was extracted from the Tafel slope, which is a significant parameter regarding to the HER dynamics. As displayed in Fig. 2b, the Tafel slope of Ni_2_P–CoOOH (118 mV dec^−1^) is consistently lower than the CF (411 mV dec^−1^) and NiCo-pre (200 mV dec^−1^), and even lower than the commercial Pt/C (131 mV dec^−1^), illustrating its robust catalytic kinetics for HER. Additionally, EIS in Fig. 2c shows that Ni_2_P–CoOOH possesses a smaller charge transfer resistance (*R*_ct_) of 25.5 Ω than NiCo-pre (50.8 Ω), indicating the accelerated electron transmission capability. The double-layer capacitance (*C*_dl_) was measured to estimate the electrochemical active surface area (ECSA), which is a considerable parameter to evaluate the catalytic performance (Figs. 2d, S9). The higher *C*_dl_ value and normalized polarization curve of Ni_2_P–CoOOH than NiCo-pre further indicate the high exposure of active sites after electrochemical driven reconstruction (Fig. S10). Therefore, the lower potential, higher current density, smaller Tafel slope, and lower resistance all suggest a superior HER performance of Ni_2_P–CoOOH, which could achieve a current density of more than 1500 mA cm^−2^ in neutral solution. Besides electrocatalytic activities, stability is also an assessed value for realizing large-scale hydrogen production, which can be obtained via multiple testing methods. Long-term chronoamperometry (*j* − *t*) test shows that the electrocatalytic HER of Ni_2_P–CoOOH is allowed to proceed for more than 100 h at a current density close to 1200 mA cm^−2^ with negligible decay (Fig. 2e). Notably, inappreciable fluctuation can be observed in local message of *j* − *t* curve, which makes it a promising material to serve as hyperstable hydrogen evolution electrocatalysts for industrial neutral water splitting. The post-test characterizations reveal no apparent changes of the morphology and crystal structure after a long-term high-current electrolysis (Fig. S11). On the other hand, multi-step chronoamperometry curves (Fig. 2f) were measured under a wide potential range (from 140 to 450 mV) and were recorded across a broad current density (from 100 to 1000 mA cm^−2^). The results showed that the electrocatalytic activities remain stable at each step during the test, confirming the excellent stability of Ni_2_P–CoOOH catalyst under mutational potentials. Contraposing different industrial requirement, it shows desirable capability suitable for lossless switchover in multi-scenario. The accelerated cyclic polarization curves (Fig. S12) for Ni_2_P–CoOOH demonstrate superior cycling stability.

To translate the high current density of Ni_2_P–CoOOH catalysts into practical device, we fabricated a proof of concept two-electrode system (Ni_2_P–CoOOH||Ni–Fe LDH) for water splitting in 1 M PBS solution (Fig. S13) [43]. The Ni–Fe layered double hydroxide (LDH) as a common anode material was used in this system (Fig. S14) [44]. For comparison, we also deposited commercial noble metal catalysts RuO_2_ and Pt/C onto CF and used these as anode and cathode (denoted as Pt/C||RuO_2_), respectively. The device characteristics are plotted in Fig. 2g, which show that the reaction for the Ni_2_P–CoOOH starts around 1.63 V and rapidly surpasses noble metal catalysts at higher current density (> 150 mA cm^−2^). To assess the catalytic stability of the system, chronoamperometry curves are recorded at 2.3 V for water splitting. As shown in Fig. 2h, the Ni_2_P–CoOOH||Ni–Fe LDH system retains its catalytic activity over 20 h at high current density of 200 mA cm^−2^, but noble metal system shows a rapid loss of catalytic activity over time. For demonstrating the advantages of the Ni_2_P–CoOOH catalyst for large-scale H_2_ production, we collected the generated H_2_ from the Ni_2_P–CoOOH||Ni–Fe LDH catalyst-driving system via a Hoffman apparatus setup (Figs. 2j, S15). Obviously, the amounts of produced H_2_ collected at current density of 200 mA cm^−2^ match well with the theoretically calculated value, corresponding to a Faradaic efficiency of ~ 96% (Fig. 2i). The systematic electrochemical studies sufficiently confirm that the multiscale coordinated regulation catalyst of Ni_2_P–CoOOH has glorious HER activity in neutral solution.

**Fig. 3:**
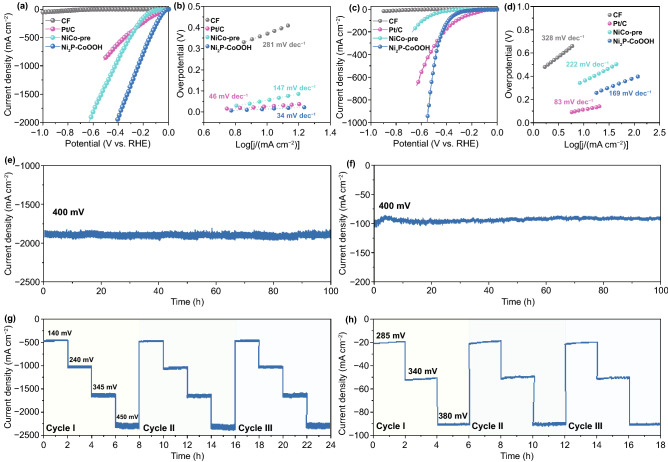
Electrocatalytic properties in 1 M KOH electrolyte and seawater. **a**, **b** Polarization curves and the corresponding Tafel plots in 1 M KOH electrolyte. **c**, **d** Chronopotentiometric curves and multi-chronoamperometric response curve in 1 M KOH electrolyte. **e**, **f** Polarization curves and the corresponding Tafel plots in seawater. **g**, **h** Chronopotentiometric curves and multi-chronoamperometric response curve in seawater

Alkaline solution is the most widespread applicable electrolyte, which has been applied in various electrocatalytic fields such as chlor-alkali industry and hydrogen manufacturing [45]. Therefore, we also measured the electrocatalytic performance in alkaline solution. As shown in Figs. 3a–d and S16, the Ni_2_P–CoOOH catalyst can achieve a high current density more than 2000 mA cm^−2^. Furthermore, there is barely any loss of catalytic activity even after 100 h of the hydrogen evolution at ~2000 mA cm^−2^ in 1 M KOH solution. In addition, water splitting device illustrates the remarkable practical ability of Ni_2_P–CoOOH catalyst, which can be directly used in industrial hydrogen production (Fig. S17). The earth-abundant seawater is considered as a potential electrolyte for catalysis. However, the poor conductivity (compared to 1 M KOH) and strong corrosively result in weak activity and instability, which remains a challenging task for electrocatalytic HER in seawater [46, 47]. As shown in Figs. 3e–h and S18, the Ni_2_P–CoOOH catalyst exhibits intriguing properties of HER in simulated seawater, surpassing all of the catalysts in previous reports, which will expedite the catalytic industrialization process of seawater. In summary, the comprehensive electrochemical studies suggest the high catalytic activity, high current density, long cycling stability of Ni_2_P–CoOOH, which may be a candidate cathode for industrial hydrogen production over a wide range of pH, even in seawater.

### Hydrophilic Characterization

**Fig. 4:**
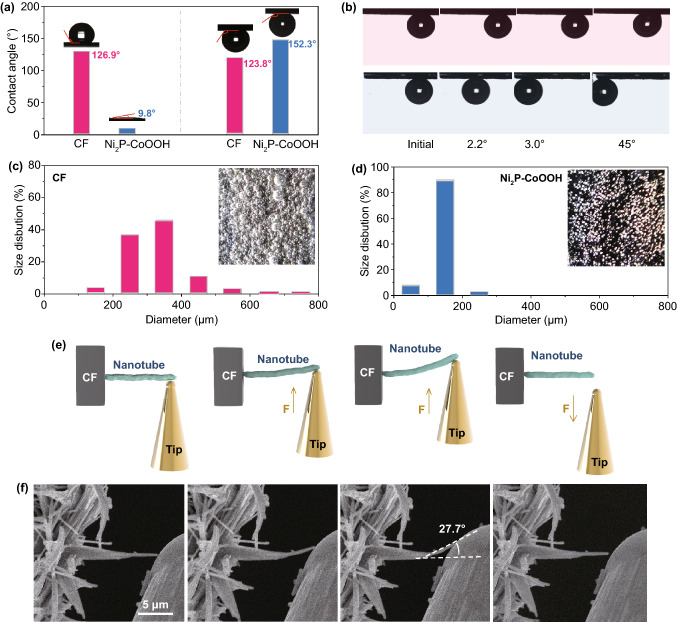
Mass transport and mechanical properties. **a** CAs of a PBS (1 M) droplet on the surfaces of catalysts (left), and CAs of a H_2_ bubble on the surfaces of the catalyst under 1 M PBS electrolyte (right). **b** Slide angle tests of catalysts. **c, d** Size distributions of H_2_ bubbles on the surfaces of a CF and Ni_2_P–CoOOH, and (inset) photographs show sharp contrast during the release of H_2_ bubbles on the surfaces of CF and Ni_2_P–CoOOH. **e, f** In situ bending deformation and restoration measurement by SEM probe

In-depth understanding the reaction kinetics of the hydrogen evolution reaction is of the essence for researchers in exploring highly efficient electrocatalysts [48, 49]. The excellent mass transfer property is widely accepted to be the crucial factor of accelerative catalytic kinetics, especially at high current density [50]. Wettability is an interaction at solid–liquid interface, which is an important parameter to evaluate the mass transfer property [51]. To quantitatively analyze the differences between the samples, we measured the contact angles (CAs) of a droplet of 1.0 M PBS on their surfaces (Fig. S19). The CAs are 130° and 9.8° for CF and Ni_2_P–CoOOH, respectively, indicating the remarkable electrolyte wettability of Ni_2_P–CoOOH (Fig. 4a, left), which benefits from porous structure and the surface hydrophilic groups. Additionally, the under-electrolyte surface wettability of the as-prepared electrode was investigated by hydrogen bubble contact angle measurements in 1 M PBS (Fig. S20). The result indicates that the surface of Ni_2_P–CoOOH has stronger adsorption of electrolyte than bubble, which further supports the above conclusions (Fig. 4a, right). Therefore, the strong interaction at solid–liquid interface accelerates liquid electrolyte transfer, which plays a significant role in getting high current density. Moreover, the generated hydrogen bubbles tend to adhere or aggregate on the surface of the catalyst, especially at high current density, resulting in decreased solid–liquid contact area and limited electron transfer [52]. Therefore, a unique “superaerophobic” characteristic is bound to promote the HER performance at high current density. The slide angle tests show a hydrogen bubble is readily pinned on the bare CF, whereas detaches with a superfast speed off the surface of Ni_2_P–CoOOH (Fig. 4b). Indeed, the surface hydrophilic groups of CoOOH nanosheets can absorb water molecules onto the electrode surface because of the strong hydrogen binding forces [53]. As a result, it reduces gas–solid interface friction and promotes the release of hydrogen bubbles from the electrocatalyst surface, which is crucial for HER at high current density. The adhered H_2_ bubbles inevitably isolate surface from electrolyte and induce deteriorated HER performance, due to blocked electrolyte transfer and consequent ineffective active sites. Therefore, the re-activation of active sites plays an important role to get the excellent catalytic activity at high current density [19]. We recorded videos to analyze the hydrogen bubble size distributions and releasing behavior on the surface, which reflects the ability to re-expose catalytic sites to the electrolyte. Clearly, hydrogen bubbles firmly adhere to the surface of bare CF and grow to larger sizes (~60% are larger than 300 µm), covering many surface catalytic sites (Fig. 4c and Video S1). In contrast, hydrogen bubbles smaller than 200 µm leave the surface of Ni_2_P–CoOOH quickly, resulting in the constant exposure of catalytic sites to the surrounding electrolyte (Fig. 4d and Video S2). According to the solid–liquid–gas interface theory, structures with roughness at both micro- and nanoscale can reduce the number of contact sites between the bubbles and catalysts, thus leading to a low interfacial adhesion and facilitating gas bubble release [54]. Apparently, all these results firmly indicate that excellent wettability plays an essential role in accelerating reaction kinetics of Ni_2_P–CoOOH and facilitating the hydrogen evolution reaction.

### Mechanical Characterization

The tensile and shaking force generated when bubbles escape and break is widely accepted to be the considerable factors of poor stability during electrocatalytic process, especially at high current density [55]. From a machinery mechanics perspective, the rich-clearance nanotubes interwoven with stacked and staggered nanosheets as “spring” can absorb the vibrational wave energy and release resilience energy, which could resist the destructive stress from surrounding environment [56, 57]. Therefore, to investigate the mechanical property of the Ni_2_P–CoOOH nanotubes, we conducted multiple bend-restoration tests for single array unit through precise control the SEM probes by mechanical arms (Fig. S21 and Video S3). Figure 4e demonstrates an operational process including bend and restoration of the Ni_2_P–CoOOH nanotube. Intuitively, the maximum angle of bending that a single nanorod can withstand is up to 27.7°. And then the nanotube can restore to initial state intact, declaring in the excellent mechanical stability. In general, such a large bending angle is prominent in all inorganic chemicals (Table S3). In conclusion, the clearances interwoven with stacked and staggered nanosheets as “buffer layer” not only endow the nanotubes high impact strength and torsion resistance but also high fatigue strength, realizing the continuous adsorption-release of external forces, and embodying the adaptive feature of catalysts.

### Charge Modulation

**Fig. 5:**
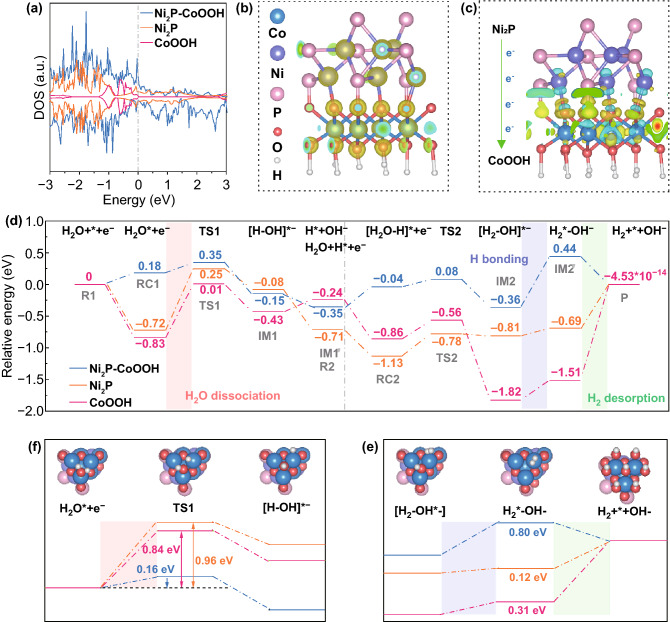
Density functional theory (DFT) calculations. **a** The calculated local density of states for Ni_2_P, CoOOH, and Ni_2_P–CoOOH. **b**, **c** Charge density difference in the interface of Ni_2_P and CoOOH. The yellow and blue isosurfaces represent charge accumulation and depletion in the space, respectively. **d** Relative energy profiles of the various reaction species along the reaction pathway, including the H* formation process (left panel) and H_2_ formation process (right panel) in neutral on the basal plane of Ni_2_P, CoOOH, and Ni_2_P–CoOOH, respectively. **e**, **f** Rate-determining step of Ni_2_P, CoOOH, and Ni_2_P–CoOOH

The effective bond breaking of H_2_O molecules is an intrinsic property for excellent HER performance, which is closely related to structural characteristics at the atomic level and the derived electron behavior [58, 59, 60]. Therefore, DFT calculations were performed to investigate carefully on interactions and the change of electronic structure in the heterointerface. The corresponding DFT calculations in Fig. 5a reveal that the Ni_2_P and CoOOH have distinct metallic properties, with the large local density of states (DOS) across the Fermi level. Furthermore, Ni_2_P–CoOOH has higher electron charge density at the Fermi level, suggesting that the interface most likely plays a role in improving the electrical conductivity. The charge density difference images reveal a strong charge redistribution at the interface and an intuitive way to trace the behavior of electrons transfer, which explicitly demonstrates the electron transfer from Ni_2_P to CoOOH (Fig. 5b, c). Obviously, the electrons of the Ni atoms prefer to flow toward the O atoms, leading to the O atoms having a more negative charge, which may promote the dissociation of H_2_O. Then, the O atoms with more electronegativity would induce charge density migration from Co atoms, which may promote the water adsorption ability at Co sites. In addition, structural information, the energy barriers of the reaction in neutral HER catalytic pathways are further calculated and displayed in Fig. 5d. Clearly, the H_2_O prefers to occupy the top site of Co by the Co–O interaction due to its endothermic manner in Ni_2_P–CoOOH. But its exothermic manner in CoOOH and Ni_2_P, determining the next step would proceed on a substantially decreased potential energy surface (RC1). Then, the cleavage of HO–H bond goes through a transition state (TS1) with an energy barrier to form an intermediate (IM1) which with H* on O site and adsorbed OH (HO*) on Co site. Specifically, Ni_2_P (0.96 eV) and CoOOH (0.84 eV) have giant water dissociation energy barriers. In contrast, the energy barrier of the Ni_2_P–CoOOH dramatically decreases to only 0.16 eV, suggesting that the change of electric charge density could lower the energy barrier of H_2_O adsorption and dissociation reaction at interface (Figs. 5e, S22). The formed IM1 is followed by desorption of HO* from the catalyst surface in an exothermic manner, leaving the vacated Co top site which is occupied by another H_2_O molecule easily, and then form another reactant complex (RC2). In the second step, a transition state (TS2) energy barrier must to be overcome, which includes the HO–H bond breaking in H_2_O*. One of the H atoms in the H_2_O* combines with the H* on the O site that produced in the first step to form an intermediate (IM2), where the generated H_2_* and HO* occupy the O site and Co site, respectively. This step is an exothermic reaction with significant energy difference, resulting in a huge difference of potential energy surface. The HO* prefers to form OH- that would rapidly migrate from the catalyst surface into the solution in an endothermic manner, leaving the H_2_*(IM2′) on the catalyst surface. It is obvious that, the energy barrier in Figs. 5f and S22 shows a value of 0.80 eV for Ni_2_P–CoOOH, indicating the desorption of OH^-^ is the rate-determining step in hydrogen evolution in neutral condition. The huge difference of adsorption strength in IM2´ is generated by the energy consumption of M–OH bond breaking. Finally, the H_2_* is spontaneously to form H_2_ gas to be eliminated. Interestingly, the hydrogen desorption reactions on O sites of Ni_2_P–CoOOH in a spontaneous manner, which shows that the transfer of electrons leads to the variation of free energy for hydrogen adsorption. Overall, the basal plane of Ni_2_P and CoOOH is almost inert for neutral HER catalysis with two rate-determining steps of water dissociation (red area) and H_2_ desorption (green area), but the only one rate-determining step (blue area) in Ni_2_P–CoOOH shows that heterointerface enables the HER catalysis to proceed on a much lower potential energy surface. The research on the catalytic pathways clearly reveals heterointerface could intrinsically boost the water dissociation and hydrogen desorption kinetics and thus endow Ni_2_P–CoOOH with exceptional neutral catalysis ability.

## Conclusion

In this conclusion, we proposed a multiscale coordinated control strategy to experimentally obtain 2D CoOOH sheet-encapsulated Ni_2_P into tubular arrays electrocatalytic system for the large-scale hydrogen production from industrial electrolysis water. By the visual contact angle test, the superaerophobic and superhydrophilic shown by the surface of 2D CoOOH sheet with burr in the array configuration will be beneficial to the good infiltration of electrolyte and slippage of hydrogen bubbles. More importantly, by means of in situ bending deformation and restoration measurement, it was revealed that the high mechanical toughness of 2D sheet as an adaptive material can buffer the shock of electrolyte convection, hydrogen bubble rupture, and evolution through the release of stress, insuring the long cycle stability. The quantitative theoretical simulation calculation further suggested the interfacial charge modulation could intrinsically boost the water dissociation and hydrogen desorption kinetics. With “one stone and three birds,” the designed electrocatalysts realize expectant 1000 mA cm^−2^-level-current-density hydrogen evolution in neutral water and 100 mA cm^−2^-level-current-density in seawater water for over 100 h, which may push the hydrogen production by electrolysis of water from laboratory to industry.

## Electronic supplementary material

Below is the link to the electronic supplementary material.

Supplementary material 1 (PDF 1489 kb)

Supplementary material 2 (MP4 18428 kb)

Supplementary material 3 (MP4 17772 kb)

Supplementary material 4 (MP4 19311 kb)
