# Supplementary material for: 2D CoOOH Sheet-Encapsulated Ni2P into Tubular Arrays Realizing 1000 mA cm−2-Level-Current-Density Hydrogen Evolution Over 100 h in Neutral Water
Source: Nanomicro Lett. 2020 Jul 2;12:140. doi: 10.1007/s40820-020-00476-4 (PMC7770877; doi:10.1007/s40820-020-00476-4)
Supplement: Supplementary file 1 — Supplementary material 1 (PDF 1489 kb) [file 40820_2020_476_MOESM1_ESM.pdf]

Supporting Information for

2D CoOOH Sheet Encapsulated Ni_2P into Tubular-Arrays Realizing 1000 mA/cm^2-Level-Current-Density Hydrogen Evolution over 100 Hours in Neutral Water

Shucong Zhang[1], Wenbin Wang[2], Feilong Hu[1], Yan Mi[1,*], Shuzhe Wang[2], Youwen Liu[2,*], Xiaomeng Ai[3], Jiakun Fang[3], Huiqiao Li[2], and Tianyou Zhai[2,*]

[1]Guangxi Key Laboratory of Chemistry and Engineering of Forest Products, and School of Chemistry and Chemical Engineering, Guangxi University for Nationalities, Nanning, Guangxi 530008, People's Republic of China

[2]State Key Laboratory of Material Processing and Die & Mould Technology, and School of Materials Science and Engineering, Huazhong University of Science and Technology, Wuhan, Hubei, 430074, People's Republic of China

[3]State Key Lab of Advanced Electromagnetic Engineering and Technology, and School of Electrical and Electronic Engineering, Huazhong University of Science and Technology, Wuhan, Hubei, 430074, People's Republic of China

*Corresponding authors. E-mail: zhaity@hust.edu.cn (Tianyou Zhai); ywliu@hust.edu.cn (Youwen Liu); miyan@gxun.edu.cn (Yan Mi)

S1 Characterization of NiCo-pre

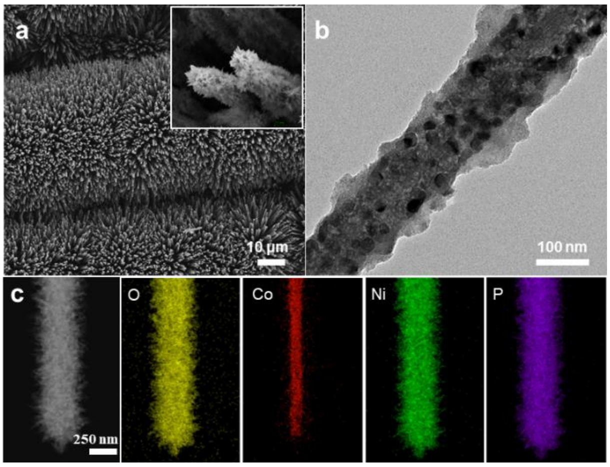

Fig. S1 Characterization of the NiCo-pre. (**a**) The SEM and high-magnified SEM images of NiCo-pre. (**b**) TEM of the NiCo-pre. (**c**) The elemental mapping of NiCo-pre shows distribution of elements. SEM shows nanowires with rough surface are highly oriented and vertically rooted on the carbon fibers with distinctive gaps between nanowires. TEM image shows many nanoparticles on the surface of

nanowires. Interestingly, the core-shell structure can be observed clearly from elemental mapping.

S2 XRD Patterns and Raman Spectrum of the Ni_2P-CoOOH and NiCo-pre

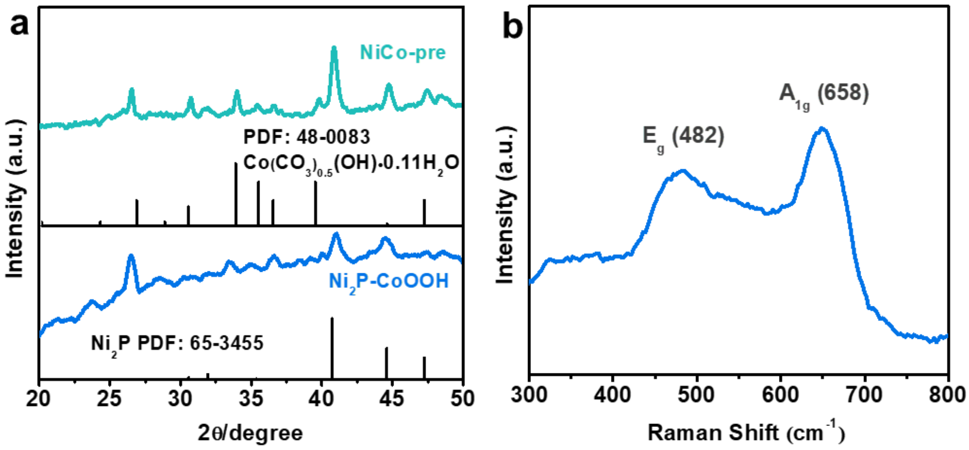

Fig. S2 XRD patterns and Raman spectrum of the Ni_2P-CoOOH and NiCo-pre. (**a**) XRD patterns of the Ni_2P-CoOOH and NiCo-pre. (**b**) Raman spectrum of the Ni_2P-CoOOH. Obviously, the XRD patterns of the NiCo-pre can be indexed to PDF#65-3544 (Ni_2P) and PDF#48-0083 ($Co(CO_3)_{0.5}(OH)·0.11H_2O$), the Ni_2P-CoOOH can be indexed to PDF#65-3544 (Ni_2P). Meanwhile, Raman spectrum suggesting existence of CoOOH in Ni_2P-CoOOH.

S3 STEM of the Interface Structure

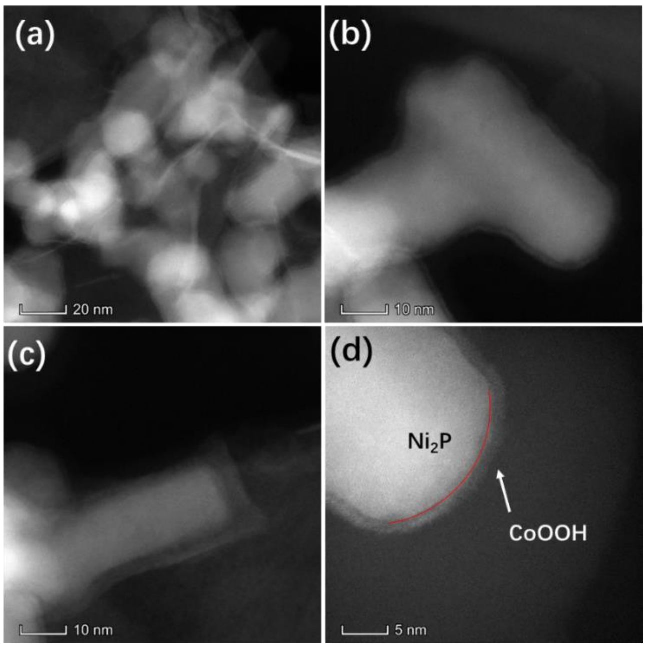

Fig. S3 STEM of the interface structure. (**a**) White particles are tightly wrapped by a translucent sheet, which indicates the formation of heterogeneous heterojunction. In the detailed STEM images in (**b, c**) the interfaces between particles and nanosheets can be observe. (**d**) Interface position was marked.

S4 Schematic of Synthetic Process

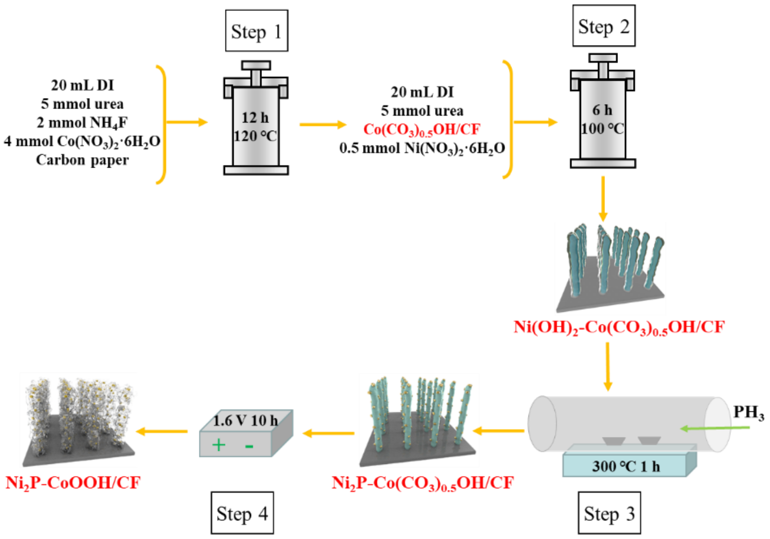

Fig. S4 Schematic of synthetic process

In the first step, $Co(CO_3)_{0.5}OH$ nanowires are grown on the surface of carbon fiber paper by hydrothermal reaction (Step 1). In the second hydrothermal reaction, a layer of nickel hydroxide is grown on the surface of $Co(CO_3)_{0.5}OH$ nanowires, forming the core-shell structure (Step 2). The layer of nickel hydroxide is further transformed into Ni_2P by a rapid low-temperature phosphating reaction. Meanwhile, the interior $Co(CO_3)_{0.5}OH$ nanowires remain unchanged, forming the Ni_2P-$Co(CO_3)_{0.5}OH$ core-shell precursor (Step 3). Subsequently, the target sample Ni_2P-CoOOH is obtained by electrochemical transformation (Step 4).

S5 Schematic Diagram of Nanotubes Formation by Electrochemical Process

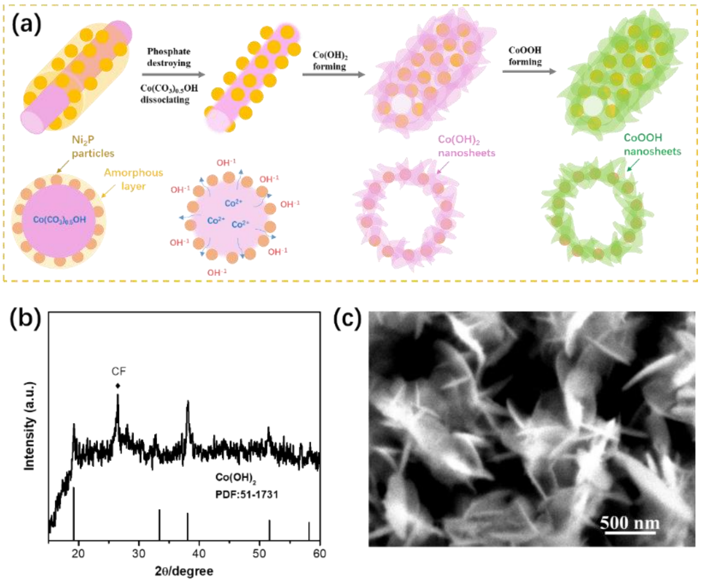

Fig. S5 (**a**) XRD and (**b**) SEM of the sample after electrochemical transformation of pure $Co(CO_3)_{0.5}OH$ nanowires for about 2 h. (**c**) Schematic diagram of a single nanotube formation by electrochemical process

From the TEM image shown in **Fig.** S1, it is obvious that the precursor is a typical core-shell structure that consist of a nanowire core and nanoparticles shell. For the $Co(CO_3)_{0.5}OH$ nanowire core, it is well known that it has a metastable-layered arrangement which with edge-sharing double octahedral chains, where M cations are located at their centers and OH- groups are located at their apices, and adjacent layers are connected by triangular $CO3^{2+}$ ions (J. Mater. Chem. A, 2016, 4, 17171-17179). When it was treated by a high current, the exterior amorphous layer was destroyed and the interior $Co(CO_3)_{0.5}OH$ was dissolved, resulting in the exudation of the internal cobalt ions through the gap between Ni_2P particles, and then formed $Co(OH)_2$ nanosheets coating on the newly exposed surface of Ni_2P particles by bonding with OH- in the electrolyte (**Fig.** S5a). We further proved this process by a short-time electrolysis of pure $Co(CO_3)_{0.5}OH$ nanowires. As shown in **Fig.** S5b-c, XRD and SEM results show that the pure $Co(CO_3)_{0.5}OH$ nanowires were transformed into $Co(OH)_2$ nanosheets after two hours of electrolysis. Following, the $Co(OH)_2$ was oxidized to CoOOH under a continuous electric current (Energy Environ. Sci., 2019, 12,739-746). Additionally, during the whole electrochemical transformation process, the external Ni_2P particles kept perfectly and connected with CoOOH nanosheets to form the nanotube structure.

S6 Line Scanning of the Ni_2P-CoOOH

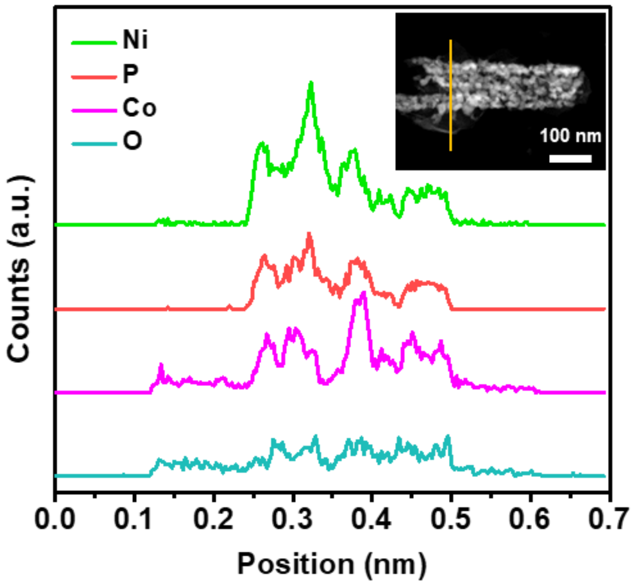

Fig. S6 Line scanning of the Ni_2P-CoOOH. The line scanning of Ni_2P-CoOOH. The result shows linear distribution of elements.

S7 XPS of the Ni_2P-CoOOH

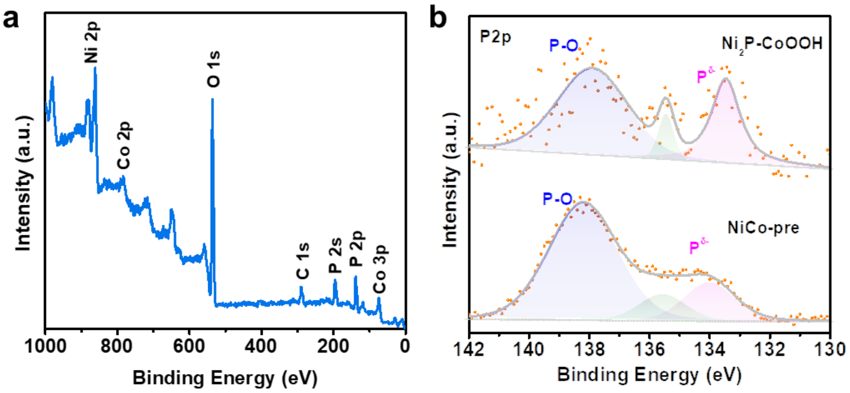

Fig. S7 XPS of the Ni_2P-CoOOH. (**a**) The wide-scanning XPS spectrum of the Ni_2P-CoOOH. (**b**) The fine scanning P 2p spectrum in Ni_2P-CoOOH

S8 Polarization Curves of Various Catalysts at Low Current Density in 1.0 M PBS Electrolyte

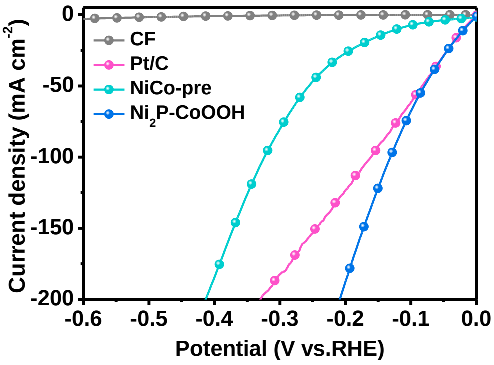

Fig. S8 Polarization curves of various catalysts at low current density with 85% IR-compensations in 1.0 M PBS electrolyte. Scan rate: 2 mV s^{-1}

S9 Voltammetry Curves of Ni_2P-CoOOH and NiCo-pre in 1.0 M PBS Electrolyte

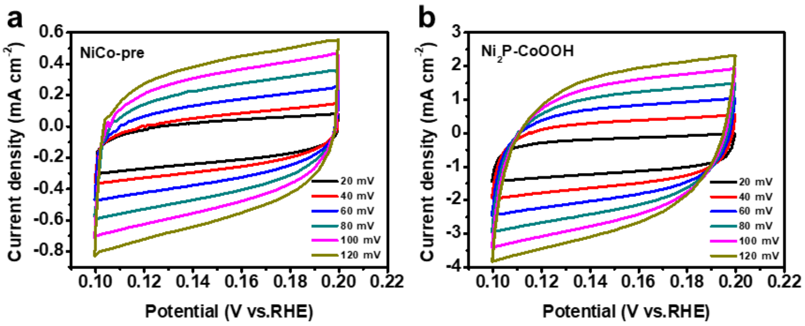

Fig. S9 Voltammetry curves of Ni_2P-CoOOH and NiCo-pre in 1.0 M PBS electrolyte. (**a**) NiCo-pre and (**b**) Ni_2P-CoOOH catalysts under the potential of 0.1~0.2 V vs. RHE at different scan rates from 20 mV s^{-1} to 120 mV s^{-1} in 1.0 M PBS electrolyte.

S10 Specific Activity of NiCo-pre and Ni_2P-CoOOH based on ECSA

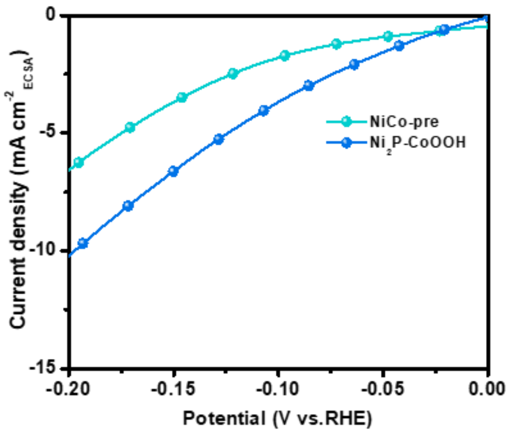

Fig. S10 Specific activity of NiCo-pre and Ni_2P-CoOOH based on ECSA in 1.0 M PBS electrolyte

S11 Characterization of Ni_2P-CoOOH after the Long-term Electrolysis in 1 M PBS Electrolyte

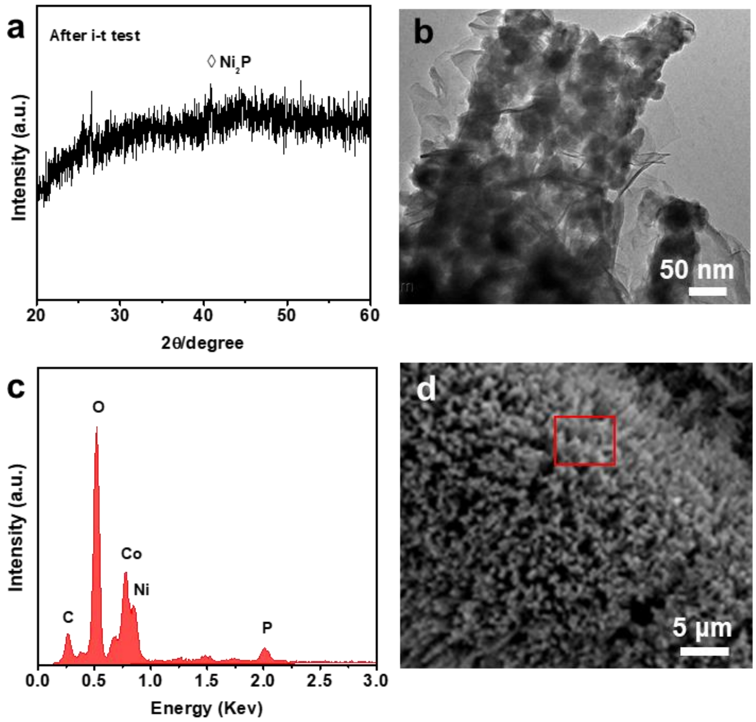

Fig. S11 Characterization of Ni_2P-CoOOH after the long-term electrolysis in 1 M PBS electrolyte. (**a-d**) XRD, TEM and EDS elemental images of Ni_2P-CoOOH after the long-term electrolysis. The XRD show the crystallinity decreased slightly. TEM for Ni_2P-CoOOH catalyst show no obvious changes of the structure. EDS shows the elements on the surface Ni_2P-CoOOH after 100 h i-t test

S12 Polarization Curves of Ni_2P-CoOOH after 5000 Cycles in 1.0 M PBS Electrolyte

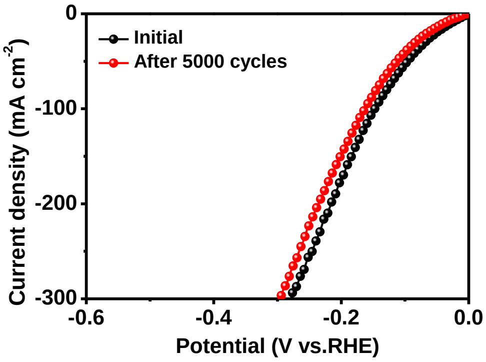

Fig. S12 Polarization curves of Ni_2P-CoOOH initially and after 5000 cycles in 1.0 M PBS electrolyte

S13 Photo of Two-electrode System

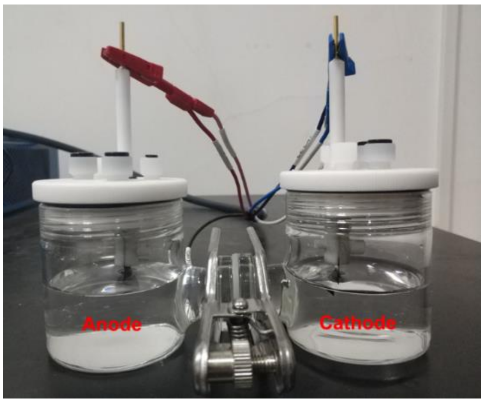

Fig. S13 The photo of two-electrode system. In this work, the H-type electrolytic cell with proton exchange membrane (PEM) was applied to prevent cathodic deposition of Pt

S14 Characterization of Ni-Fe LDH

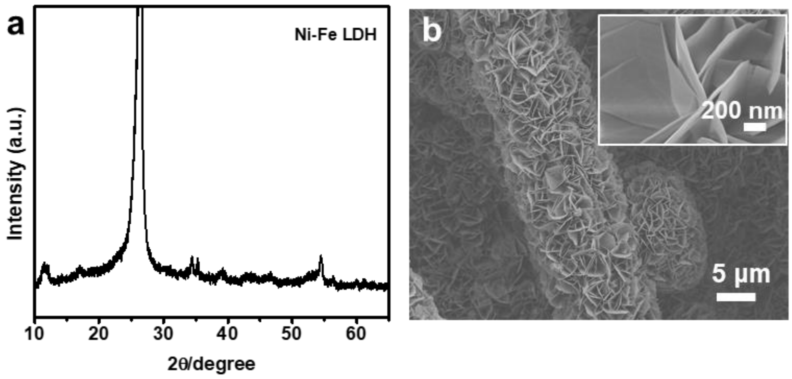

Fig. S14 (**a**) XRD pattern (**b**) SEM images of the Ni-Fe LDH obtained at 120 ºC for 6 h via a hydrothermal method

S15 Digital Photo of Hoffman Apparatus

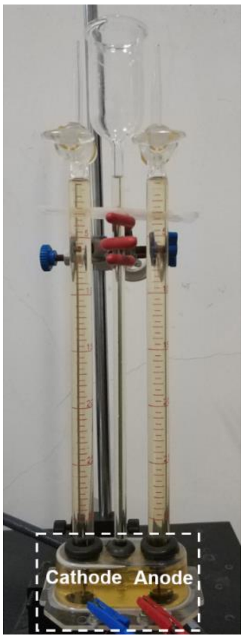

Fig. S15 The digital photo of Hoffman apparatus

S16 Electrocatalytic Properties of the Electrodes for HER in 1 M KOH Electrolyte

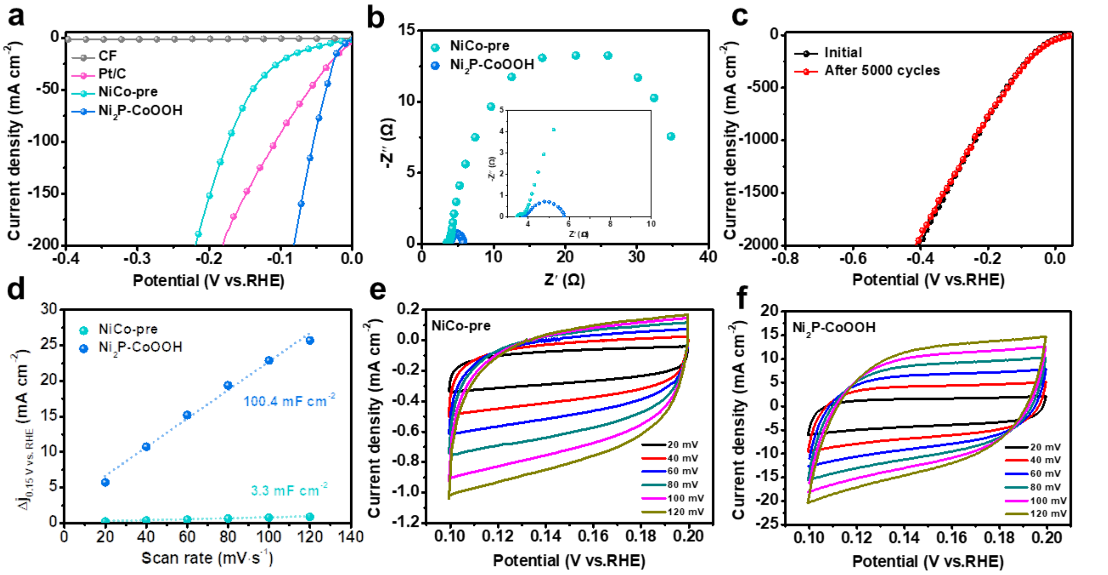

Fig. S16 Electrocatalytic properties of the electrodes for HER in 1 M KOH electrolyte. (**a**) Polarization curves of various catalysts in low current density with 85% iR-compensations. Scan rate: 2 mV s^{-1}. (**b**) EIS of Ni_2P-CoOOH and NiCo-pre at 0.2V vs. RHE. (**c**) Polarization curves of Ni_2P-CoOOH initially and after 5000 cycles. (**d**) The double-layer capacitance (C_{dl}) is determinated by the slope of the fitting line. Voltammetry curves of (**e**) NiCo-pre and (f) Ni_2P-CoOOH catalysts under the potential of 0.1~0.2 V vs. RHE at different scan rates from 20 mV s^{-1} to 120 mV s^{-1} in 1.0 M KOH electrolyte

S17 Overall Water Splitting in 1.0 M KOH Electrolyte

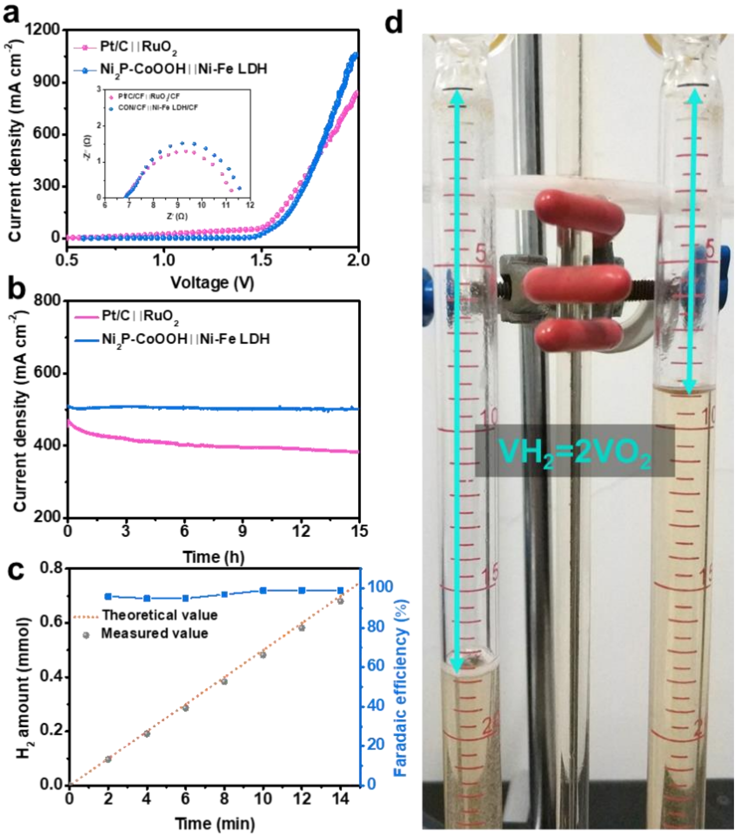

Fig. S17 Overall water splitting in 1.0 M KOH electrolyte. (**a**) Polarization curves for overall water splitting at a scan rate of 2 mV s^{-1}. The Nyquist plots is shown in the inset**.** (**b**) Chronopotentiometric curves at a voltage of 1.8 V. (**c**) H_2 amount for Ni_2P-CoOOH ||Ni-Fe LDH at a fixed current density (**d**) optical picture of the measured setup of the Hoffman apparatus

S18 Electrocatalytic Properties of the Electrodes for HER in Seawater

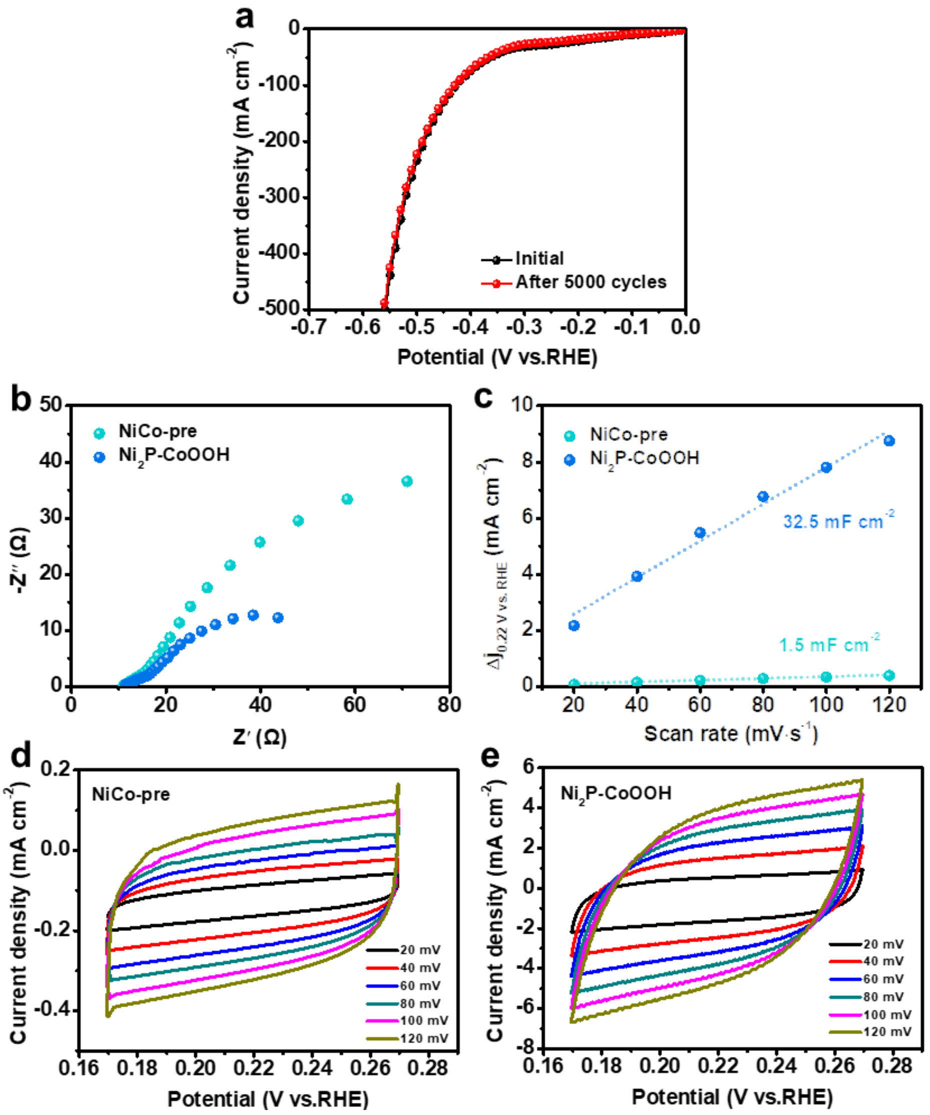

Fig. S18 Electrocatalytic properties of the electrodes for HER in seawater. (**a**) Polarization curves of Ni_2P-CoOOH initially and after 5000 cycles. (**b**) **EIS** of Ni_2P-CoOOH and NiCo-pre at 0.2V vs. RHE. (**c**) The double-layer capacitance (C_{dl}) is determinated by the slope of the fitting line. Voltammetry curves of (**d**) NiCo-pre and (**e**) Ni_2P-CoOOH catalysts under the potential of 0.17~0.27 V vs. RHE at different scan rates from 20 mV s^{-1} to 120 mV s^{-1} in seawater

S19 Contact Angles Measurements

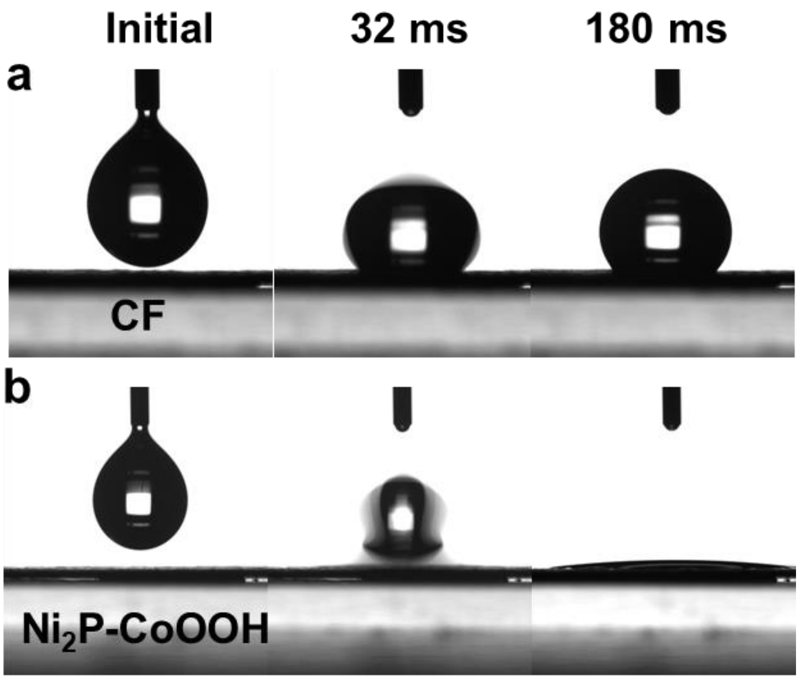

Fig. S19 Contact angles measurements. The contact angles (CAs) of a droplet of 1.0M PBS on the surface of (**a**) CF and (**b**) Ni_2P-CoOOH.

S20 Hydrogen Bubble Contact Angles Measurements

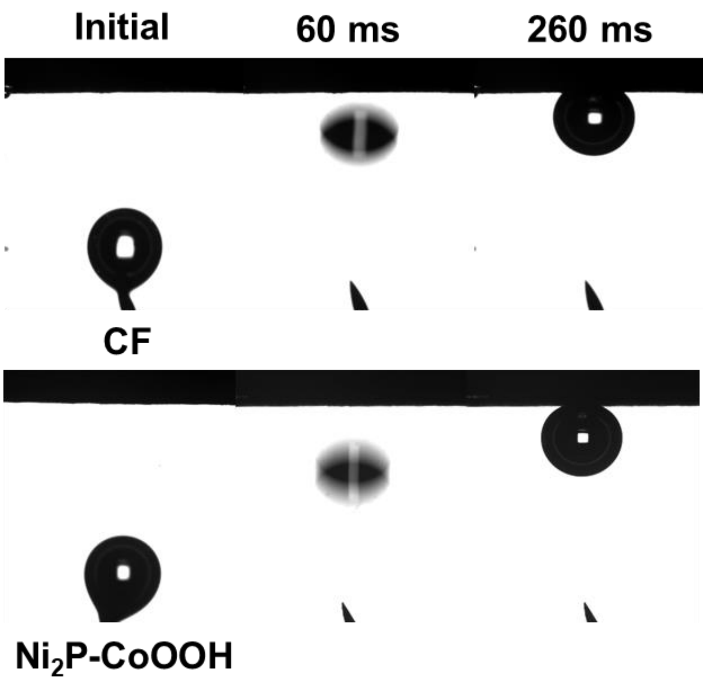

Fig. S20 Hydrogen bubble contact angles measurements. The hydrogen bubble contact angles measurements of (**a**) CF and (**b**) Ni_2P-CoOOH under 1M PBS electrolyte

S21 *In situ* Bending Deformation and Restoration Measurement by SEM Probe

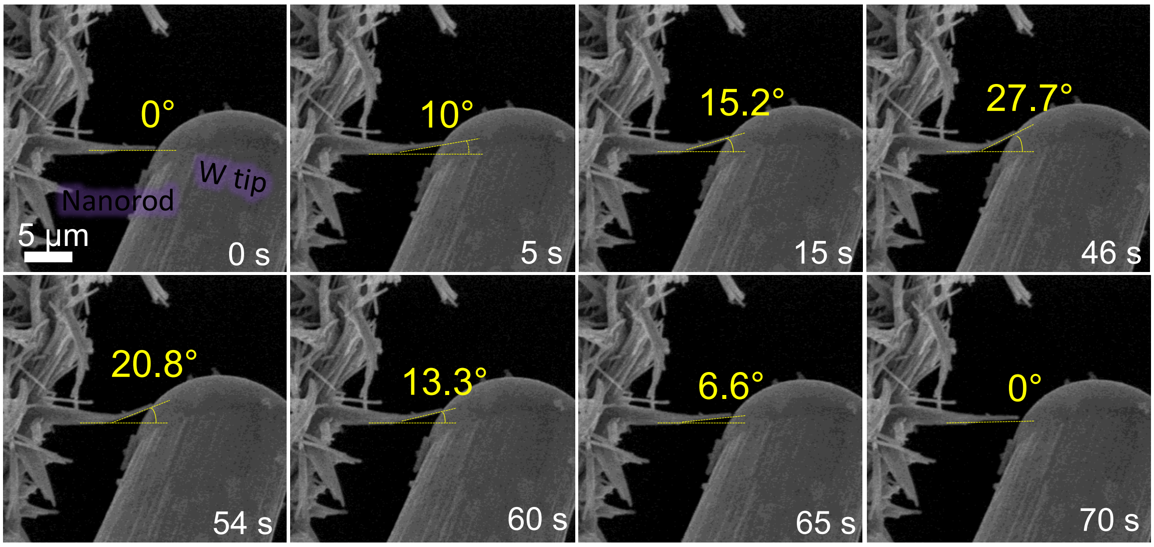

Fig. S21 *In situ* bending deformation and restoration measurement by SEM probe

S22 All Structures for These Two Key Steps for Ni_2P and CoOOH

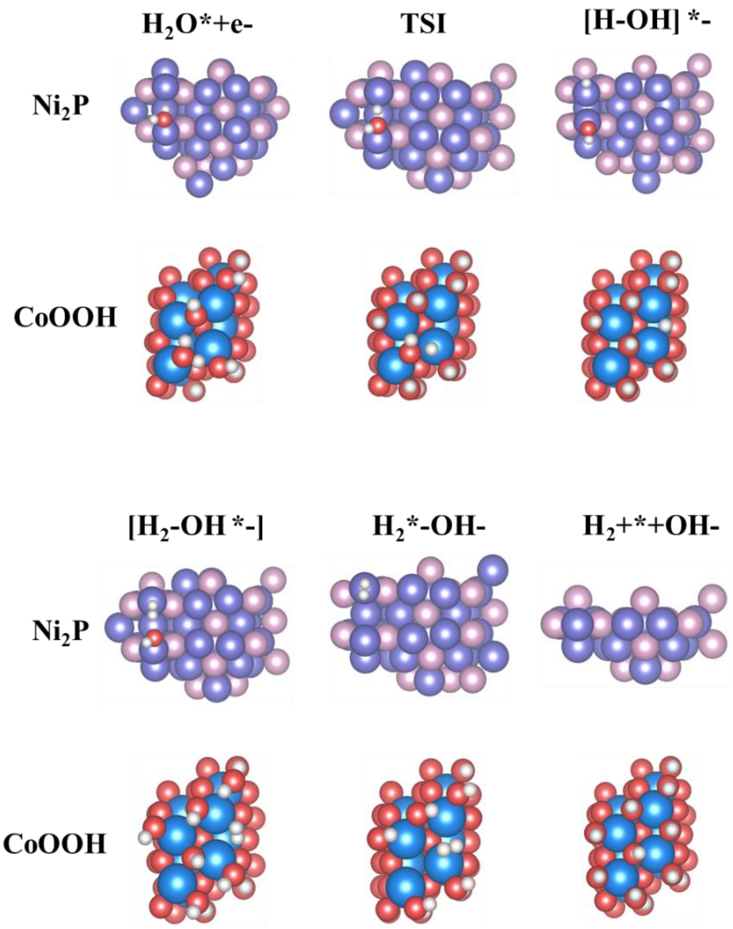

Fig. S22 All structures for these two key steps for Ni_2P and CoOOH

Table S1 Electrocatalytic HER properties for other reported products catalysts in 1M PBS electrolyte

Catalysts	Electrolytes	η_{10} (mV)	Stability test	References
**Ni_2P-CoOOH**	**1.0 M PBS**	**20**	**1200 mA cm^2 @100 h**	This work
Pt/np-$Co_{0.85}Se$	1.0 M PBS	55	10 mA cm^2 @40 h	Nat. Commun. 2019, 10,1743
CoMoNiS-NF-31	1.0 M PBS	107	10 mA cm^2 @20 h	J. Am. Chem. Soc. 2019, 141, 10417−10430
CoP/NiCoP/NC	1.0 M PBS	123	10 mA cm^2 @80 h	Adv. Funct. Mater. 2018, 1807976
FLNPC@ MoP-NC/MoP-C/CC	1.0 M PBS	103	10 mA cm^2 @50 h	Adv. Funct. Mater. 2018, 28, 1801527
$Ni_{0.1}Co_{0.9}P$	1.0 M PBS	125	30 mA cm^2 @20 h	Angew. Chem. Int. Ed. 2018, 130,15671–15675
$Ni_{0.89}Co_{0.11}Se_2$ MNSN/NF	1.0 M PBS	82	~40 mA cm^2 @40 h	Adv. Mater. 2017, 29, 1606521
CoP NW/Hb	1.0 M PBS	121	~60 mA cm^2 @100 h	Nano Research 2017, 10, 1010.
$NiCo_2P_x$	1.0 M PBS	63	20 mA cm^2 @30 h	Adv. Mater. 2017, 29, 1605502
SiO2/PPy NTs-CFs	1.0 M PBS	70	~55 mA cm^2 @30 h	Angew. Chem. Int. Ed. 2017, 56,8120–8124
**Co-HNP**	**1.0 M PBS**	**87**	**150 mA cm^2 @20 h**	**Angew. Chem. Int. Ed. 2016, 55, 6725.**

Table S2 Electrocatalytic HER properties for other reported products catalysts in seawater

Catalysts	Electrolytes	η_{10} (mV)	Stability test	References
Ni_2P-CoOOH	Seawater	194 mV	100 mA cm^{2} @100 h	This work
NiCoP/NF	Seawater	287 mV	10 mA cm^{2} @20 h	ACS Appl. Energy Mater. 2019, 2, 3910−3917
CoMoP@C	Seawater	450 mV	20 mA cm^{2} @10 h	Energy Environ. Sci., 2017, 10, 788--798
Cobalt Selenide	Seawater	350 mV	—	Adv. Energy Mater. 2018, 8, 1801926
**U-CNT-900**	**Seawater**	**670 mV**	**12 mA cm^{2} @7 h**	**Nanoscale, 2015, 7, 2306–2316**

Table S3 Comparison on the maximum angle of bending of 1D inorganic nano-materials with bending method

Materials	Angle range	Reference
**Ni_2P-CoOOH nanowires**	**≤27.7°**	**This work**
Au nanowires	≤25.8°	Nat. Mater. 2005, 4, 525-529
Carbon nanotubes	≤26.8°	Adv. Mater. 2000, 12, 1295-1298
ZnO nanowires	≤30.7°	Phys. Rev. Lett. 2008, 101, 175502.
SiC nanorods	≤41.0°	Science 1997, 227, 1971-1975
Carbon nanotubes	≤27.9°	Phys. Rev. Lett. 2000, 85, 622-625
TiO_2 nanowires	≤12.8°	Mat. Sci. Eng. A 2015, 641, 281-289
Si nanowires	≤18°	Nano Lett. 2006, 6, 622-625
ZnO nanowires	≤20°	Mater. Res. Express 2019, 6, 025012
